# Early Origins of Divergent Patterns of Morphological Evolution on the Mammal and Reptile Stem-Lineages

**DOI:** 10.1093/sysbio/syac020

**Published:** 2022-03-11

**Authors:** Neil Brocklehurst, David P Ford, Roger B J Benson

**Affiliations:** Department of Earth Sciences, University of Cambridge, Downing Street, Cambridge, UK; Department of Earth Sciences, University of Oxford, South Parks Road, CB2 3EQ Oxford, UK; Department of Earth Sciences, University of Oxford, South Parks Road, CB2 3EQ Oxford, UK

## Abstract

The origin of amniotes 320 million years ago signaled independence from water in vertebrates and was closely followed by divergences within the mammal and reptile stem lineages (Synapsida and Reptilia). Early members of both groups had highly similar morphologies, being superficially “lizard-like” forms with many plesiomorphies. However, the extent to which they might have exhibited divergent patterns of evolutionary change, with the potential to explain the large biological differences between their living members, is unresolved. We use a new, comprehensive phylogenetic dataset to quantify variation in rates and constraints of morphological evolution among Carboniferous–early Permian amniotes. We find evidence for an early burst of evolutionary rates, resulting in the early origins of morphologically distinctive subgroups that mostly persisted through the Cisuralian. Rates declined substantially through time, especially in reptiles. Early reptile evolution was also more constrained compared with early synapsids, exploring a more limited character state space. Postcranial innovation in particular was important in early synapsids, potentially related to their early origins of large body size. In contrast, early reptiles predominantly varied the temporal region, suggesting disparity in skull and jaw kinematics, and foreshadowing the variability of cranial biomechanics seen in reptiles today. Our results demonstrate that synapsids and reptiles underwent an early divergence of macroevolutionary patterns. This laid the foundation for subsequent evolutionary events and may be critical in understanding the substantial differences between mammals and reptiles today. Potential explanations include an early divergence of developmental processes or of ecological factors, warranting cross-disciplinary investigation. [Amniote; body size; constraint; phylogeny; rate.]

Amniotes, the terrestrialized vertebrates, are a diverse group comprising more than 25,000 living species. Their earliest fossils occur around 318 million years ago and already include representatives of the two major subgroups that persist to the present day (Carroll 1964; Reisz 1972): Synapsida (mammal-line amniotes) and Reptilia, or Sauropsida (the stem-lineage of reptiles, including birds; hereafter referred to as Reptilia or “reptiles”). The earliest members of both groups were extremely similar in their general morphology, being small and superficially lizard-like insectivores with sprawling limb orientations. However, they rapidly radiated into a substantial ecomorphological diversity, including diversification of diets (Sues and Reisz 1998; Brocklehurst and Benson 2021), body sizes (Laurin 2004; Reisz and Fröbisch 2014; Brocklehurst et al. 2016; Brocklehurst and Brink 2017; Brocklehurst and Fröbisch 2018; Brocklehurst et al. 2020), habitat use (e.g., arboreality; Spindler et al. 2018; Mann et al. 2021), and diel activity patterns (Angielczyk and Schmitz 2014; Ford and Benson 2019). Their success has been attributed to a number of evolutionary innovations, including musculoskeletal adaptations that freed the skull from its role in lung ventilation, allowing greater skull versatility (Frazzetta 1968; Janis and Keller 2001), the evolution of temporal fenestration facilitating muscle attachment (Frazzetta 1968; Werneburg 2019; Abel and Werneburg 2021), and the evolution of the amniotic egg (Romer 1957; Carroll 1970).

Early amniotes provide a classic example of diversification following adaptive zone invasion, and various studies have sought to characterize macroevolutionary patterns during this transition, with suggestions of little substantial change in rate or mode of morphological evolution of body size or general anatomy at the origin of amniotes (Laurin 2004; Ruta et al. 2006, 2018) but substantial increases in the functional disparity of the feeding apparatus (Anderson et al. 2013) and in rates of tooth and jaw evolution (Brocklehurst and Benson 2021). However, understanding the early radiation within amniotes is less well-characterized. Analyses so far have been conducted at various phylogenetic scales, including in larger analyses of tetrapod evolution that contain a more limited sampling of early amniotes (e.g., Laurin 2004; Ruta et al. 2006, 2018; Anderson et al. 2013), as well as restricted examinations of early amniote subgroups (Brocklehurst 2016, 2017; Brocklehurst and Brink 2017; Romano et al. 2017, 2018; MacDougall et al. 2019). Studies have also focused on different portions of the anatomy, including limbs (Ruta et al. 2018), jaws and teeth (Anderson and Friedman 2013; Brocklehurst and Benson 2021), vertebrae (Jones et al. 2018, 2021), and body size (Laurin 2004). However, thus far there has been no study of macroevolutionary patterns during the origin and early radiation of amniotes including a broad selection of all clades, allowing direct comparison of the evolutionary patterns within the major lineages, and across many anatomical regions.

Discrete character state matrices provide observations of morphological variation from across the skeleton that may be used for large-scale macroevolutionary analyses. We present a new phylogenetic dataset, substantially expanded from the most recent phylogenetic assessment of early amniote evolution (Ford and Benson 2020), including species and relevant anatomical variation from across all clades spanning the Carboniferous until the end of the early Permian. We use this to assess rates of evolution and evolutionary constraints during the earliest radiation of amniotes across their anatomy and within different partitions, examining differences between early synapsids and early reptiles.

## Materials and Methods

### Dataset

We analyze a new phylogenetic dataset of early amniotes, focused on the coverage of late Carboniferous (Pennsylvanian) and early Permian (Cisuralian) taxa, and lineages that survived into the middle Permian (Guadalupian), including Therapsida and Neodiapsida. We attempted comprehensive coverage of phylogenetically informative characters from previous studies and our own observations (e.g., Benson 2012; Modesto et al. 2014), expanded from the analysis of Ford and Benson (2020) to achieve a broader sample of Paleozoic amniote lineages. To this end, we added 31 new taxa, mostly pelycosaurian-grade synapsids, moradisaurine captorhinids, and acleistorhinid parareptiles. A total of 72 additional characters were added, mostly sourced from Benson (2012) and Modesto et al. (2014). The final dataset contains 98 taxa and 366 characters (Supplementary Data 1 and 2 available on Dryad at http://dx.doi.org/10.5061/dryad.9ghx3ffj5).

Our study also includes analyses of a dataset of 144 dental traits in 534 taxa, taken from Brocklehurst and Benson (2021). This was included to evaluate variation in dental traits because it contains a greater sampling of dental characters from both jaws and the palate, being designed to investigate macroevolutionary patterns within feeding apparatus. It also contains a greater sampling of taxa, both within the interval of study and subsequent times until the Early Triassic, allowing analysis over a longer time duration than available for our primary matrix (Supplementary Data 3 available on Dryad).

### Fossilized Birth–Death Analysis

We used Bayesian phylogenetic inference under a relaxed Mkv model of character state evolution with a fossilized birth–death (FBD) tree prior (Heath et al. 2014) to infer a time-scaled phylogeny and rates of character state evolution. To account for the uncertainty in the time of the first appearances, the ages of taxa were represented by a uniform probability distribution covering the full uncertainty of the age of the formation or assemblage zone in which they first appear (see Supplementary Data 20 available on Dryad for the source of formation ages). Net speciation rate (diversification) was drawn from a uniform prior, with net extinction rate (turnover) and relative fossilization rate drawn from beta priors. Temporal variation in these parameters was not modeled. An independent gamma rates model was employed to account for rate heterogeneity between branches (an uncorrelated clock model where rates are drawn from a gamma distribution). Rate heterogeneity between characters was also modeled as a gamma distribution. The analysis was carried out with two runs containing four chains for 50 million generations, sampling every 1000, with 25$\%$ of trees discarded as burn-in. The maximum clade credibility tree was used as the phylogenetic framework for subsequent analyses. The analysis was implemented in MrBayes 3.2.6 (Ronquist and Huelsenbeck 2003).

### Analysis of Rates

Rates of character change along each branch were drawn from the results of the FBD analysis (Supplementary Data 4 available on Dryad). Variation in the rates of evolution through time was assessed by time slicing the tree at intervals of one million years between 320 and 272 Ma (from the origin of amniotes until the end of the Cisuralian). The rates of all branches crossing each time slice (not including non-amniote outgroups) were collated, and the median rate of each time slice was calculated. To assess long-term trends in rate variation, a Loess regression curve was fitted to the median rate values through time.

### Analysis of Constraint

Variation in the strength of evolutionary constraint among lineages was assessed by comparing patristic distances and morphological dissimilarities between pairs of taxa, expanding on a procedure designed by Brocklehurst et al. (2021) to assess character state saturation, or exhaustion: the point where a further evolutionary change in morphology (i.e., increasing patristic morphological distance) no longer results in an increase in the differences between taxa (i.e., morphological dissimilarity, or disparity) but instead explores a pre-established character state space, with a high prevalence of homoplasy. We indexed the morphological dissimilarity between pairs of taxa as the proportion of character scores that differ between them, calculated in the R v3.6.1 (R Core Team 2019) in the package Claddis (Lloyd 2016) using the MORD distance metric. Evolutionary change (patristic morphological distance) is represented by the total phylogenetic branch length between a pair of taxa, representing the number of character state changes that evolved since divergence from their common ancestor. To calculate this, the character/taxon matrix was reanalyzed in MrBayes using an Mkv model of character state evolution, with no information on taxon ages, constraining the topology to that found by the FBD analysis (for our primary matrix) or a composite tree representing consensus from the literature (for our additional dental matrix, see Brocklehurst and Benson 2021). This Mkv analysis produced a phylogeny in which branch lengths correspond to the inferred amount of morphological character state change (Supplementary Data 5 available on Dryad). The summed branch lengths between pairs of taxa were then used as patristic morphological distances, extracted using the R package adephylo (Jombart et al. 2010).

In general, morphological dissimilarity should increase with evolutionary state changes (i.e., with increasing patristic morphological distance). However, this increase begins to asymptote at higher patristic distances (Wagner 2000), indicating a lack of further exploration of novel character state space. This occurs because homoplastic state changes and reversals can cause increases in similarity, and homoplasy becomes more frequent with increasing patristic distance under constrained evolution (Brochu 1997; Wagner 2000). This results in character state saturation (Foote 1994), or exhaustion (Wagner 2000), whereby further increases in patristic morphological distance between taxon pairs do not, on average, lead to the greater morphological dissimilarity between them. Character state saturation, indicated by the asymptote of the relationship between morphological dissimilarity and patristic morphological distance, occurs at lower morphological dissimilarity when constraints are strong and higher dissimilarity when constraints are weak (Wagner 2000).

Individual groups were assessed for significant increases or decreases in constraint using the procedure of Brocklehurst et al. (2021), in which a Michaelis–Menten curve was fit to the comparisons of the patristic morphological distances and pairwise morphological dissimilarities between all pairs of taxa within that clade, the $V_{\max}$ (asymptote) parameter of that curve being used to represent the point of character state saturation. The significance of differences in $V_{\max}$ between portions of the phylogeny was evaluated by comparison to expectations given a uniform model of evolution, as described in Brocklehurst et al. (2021). This was implemented by simulating null character/taxon matrices under an equal rates model, with missing data scores added in the same location as in the empirical dataset. Null morphological dissimilarities between the taxa were calculated from these matrices as described above, which were compared with the patristic distances again by fitting a Michaelis–Menten curve, showing whether the clade under study reached character state saturation at a higher or lower level than in the null simulations. Character state saturation was assessed in both reptiles and synapsids, restricting the comparisons using the primary matrix to pairs of taxa that diverged within the interval of time under study: the Carboniferous–early Permian. We omitted younger branches because they were incompletely sampled and had only been included to ensure coverage of branches that originated in the early Permian, including some that continued into later intervals. However, our analysis of the dental dataset of Brocklehurst and Benson (2021) (Supplementary Data 6 available on Dryad) extends up to the early Triassic allowing insights into important later events such as the diversification of Therapsida, ankyromorph parareptiles, and Neodiapsida, at least for dental characters.

We expand on this method here to allow the detection of variation in the strength of constraint in the absence of prior hypotheses regarding the phylogenetic location of shifts. This was done by applying the method to every node in the phylogeny, comparing the patristic morphological distances and pairwise dissimilarities as described above, by fitting a Michaelis–Menten curve to estimate the $V_{\max}$ parameter and 84$\%$ confidence intervals around it. We then compared this to the null distribution resulting from the analysis of null character/taxon matrices resulting from 1000 iterations of our simulation approach. Significance was determined when the 84$\%$ confidence interval of a node’s observed $V_{\max}$ value lay entirely above the 84$\%$ quantile of the null simulations (significant release in constraint), or entirely below the 84$\%$ quantile of the null simulations (significant strengthening of constraints). We used 84$\%$ intervals because they are expected to overlap 95$\%$ of the time when two distributions are statistically identical, therefore representing a significance threshold of 0.05 (Payton et al. 2003). In contrast, two statistically identical 95$\%$ intervals will overlap 99$\%$ of the time, resulting in an increased frequency of false negatives. The entire process is carried out in R using custom code (Supplementary Data 7 and 8 available on Dryad) written using functions from the packages Adephylo (Jombart et al. 2010), Claddis (Lloyd 2016), and Phytools (Revell 2012).

### Analyses of Character Partitions

Constraints within anatomical partitions were investigated by analyzing subsets of the full character list of each analysis, representing (i) the skull (including lower jaw and mandible), (ii) postcranium, (iii) snout (antorbital region of the skull, not including palate), (iv) temporal (postorbital region of the skull), and (v) dentition (including palatal dentition; and supplemented by analysis of the more extensive dataset of dental traits from Brocklehurst and Benson 2021) (Supplementary Data 9–13 available on Dryad). For analyzing rates within the partitions, characters from each were subjected to an FBD analysis, with node ages and topology constrained to those identified by the FBD analysis of the whole dataset. This produces a tree identical to the MCC tree from analysis of the whole dataset, but where the rate values represent only those of the character partition (Supplementary Data 14–18 available on Dryad). For the analysis of constraints, characters from each partition were subjected to an undated Bayesian analysis constraining the tree to that identified by the FBD analysis of the whole dataset. This produces a tree whose branch lengths represent only character changes within the relevant partition. Rates of evolution and character state saturation were then assessed as described above.

### Analysis of Body Size

Differences in patterns of trait evolution between reptiles and synapsids may result from the macroevolutionary effects of large body sizes, which evolved frequently among early synapsids but rarely among early reptiles (Modesto et al. 2015; Brocklehurst and Brink 2017; Brocklehurst and Fröbisch 2018). To evaluate this, taxa were assigned to one of the four size categories, each representing an order of magnitude (small: $<$1 kg, medium: 1–10 kg, large: 10–100 kg, very large: 100–1000 kg) (Supplementary Data 19 available on Dryad). Using discrete categories of body size is less precise than estimates of size using continuous measurements (e.g., Alroy 1998; Campione and Evans 2012; Brocklehurst and Brink 2017; Benson et al. 2018). Nevertheless, we use discrete body size categories here because they allow the inclusion of the maximum number of taxa, including those too fragmentary to make precise mass estimates. Constraint within each size class was assessed as described above, comparing pairs of taxa within each size category, limiting comparisons to pairs of taxa that diverged during the Carboniferous–early Permian.

To examine how the size evolution varied between synapsids and reptiles, four models of discrete character evolution were fit to the phylogeny and body size categories: single regime, equal rates (all possible transitions between character states have a single rate, and the rate is consistent between reptiles and synapsids); single regime, all rates different (all possible transitions between character state may a have a different rate, but the rates are consistent between reptiles and synapsids); multi regime, equal rates (all possible transitions between character state have a single rate, but the rate varies between reptiles and synapsids); multi regime, all rates different (all possible transitions between character state may a have a different rate, and the rates vary between reptiles and synapsids). The model fitting was carried out using the *fitMultiMk* function in phytools (Revell 2012). The fit of the models to the data was assessed using the Akaike weights.

## Results

### Phylogeny

**Figure 1.**
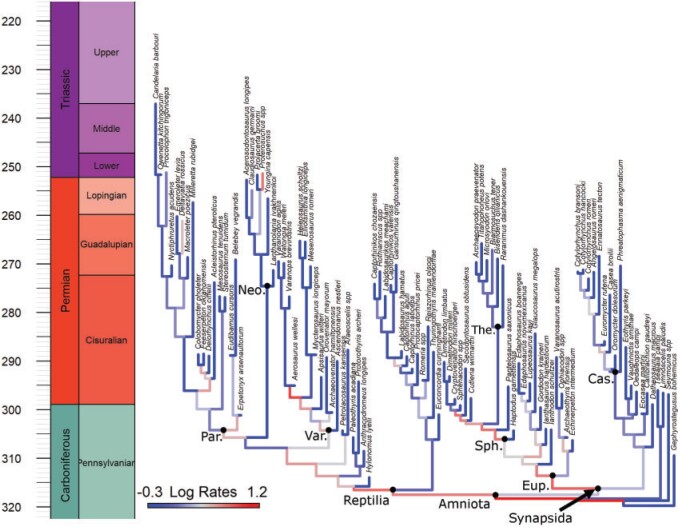
The maximum clade credibility tree produced from the fossilized birth–death analysis, with branches color coded according to log-transformed evolutionary rates. Nodes discussed in the text labeled: Eup., Eupelycosauria; Var., Varanopidae; Par., Parareptilia; Sph., Sphenacodontia; Cas., Caseidae; Ther., Therapsida; Neo., Neodiapsida.

Our phylogenetic analysis returns a tree topology that is consistent with that found by Ford and Benson (2020), in spite of the addition of characters and taxa (Fig. 1 and Fig. S2 of the Supplementary material available on Dryad). Parareptiles are found as the sister to neodiapsids and varanopids are found as reptiles, suggesting that support for this contentious phylogenetic hypothesis (e.g., Benoit et al. 2021; Bazzana et al. 2021) remains high even given a larger sample of early synapsids. The addition of further pelycosaurian-grade synapsids produced results broadly consistent with recent analyses of this grouping (Brocklehurst and Fröbisch 2018; Maddin et al. 2020; Berman et al. 2020). The only noticeable discrepancy is that *Eocasea, Callibrachion*, and *Datheosaurus* are found as outgroups to other caseasaurs (Eothyrididae and Caseidae) rather than being within caseids (as found by Reisz and Fröbisch 2014; Brocklehurst et al. 2016; Berman et al. 2020).

### Analysis of Rates

**Figure 2.**
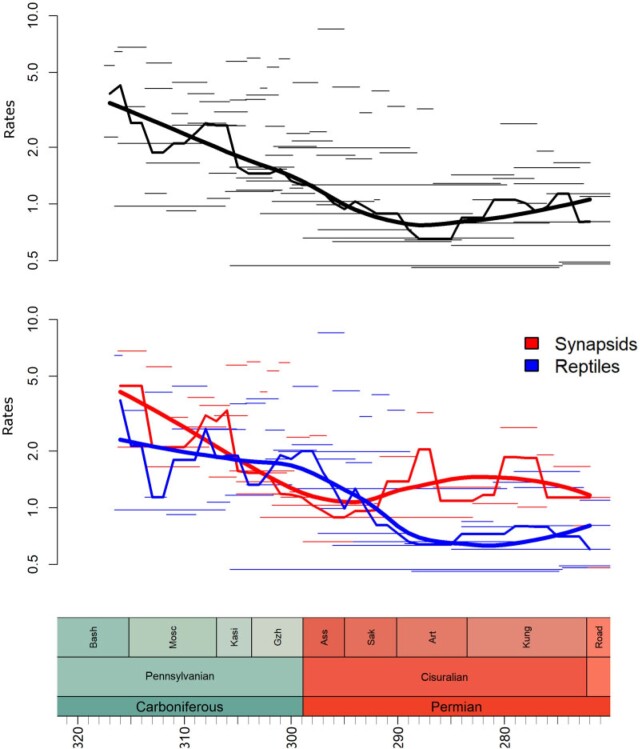
Rates of evolution through time. Narrow lines represent the rates of individual branches. The mid-weight line represents the median rate of each 1 million year time slice. The thick line represents Loess-fitted regression between median rate and time. (a) All amiotes and (b) reptiles and synapsids were compared.

Rates were highest during the earliest history of amniotes, decreasing through the late Carboniferous until the middle of the early Permian (Cisuralian) (Figs. 1 and 2a). Both synapsids and reptiles exhibit high evolutionary rates that decreased through time during the late Pennsylvanian/earliest Cisuralian. However, rates of evolution in synapsids remain about twice as high as those of reptiles through the latter half of the Cisuralian (Fig. 2b). Among subclades, rates are highest in Eupelycosauria (among synapsids) and during the early divergences of varanopids and parareptiles (among reptiles) (Fig. 1).

### Analysis of Constraints

**Figure 3.**
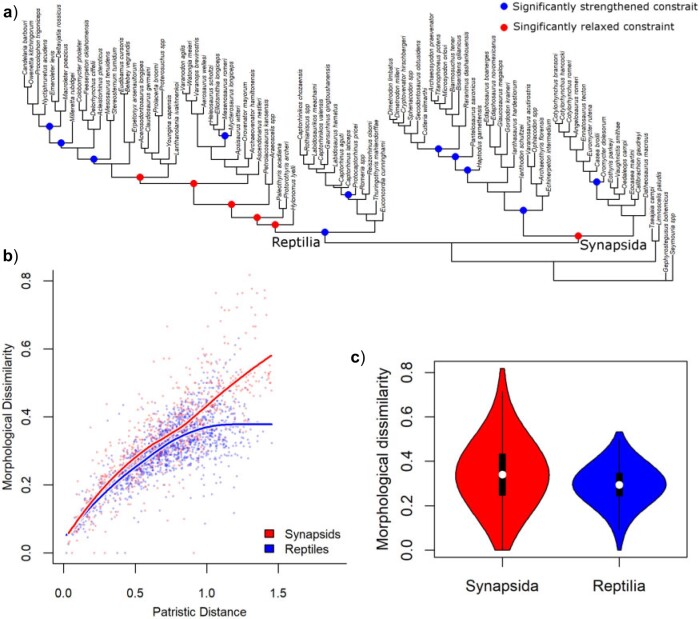
Patterns of constraint in amniotes. (a) Significant variation in constraint plotted over a phylogeny where branch lengths represent the amount of character change along the branch. Nodes in red experience a significant relaxation of constraint. Coloured nodes indicate significant strengthening or relaxation of constraints within clade. (b) Comparison of patristic distances and morphological dissimilarity in synapsids (red) and reptiles (blue). Each point represents a pairwise comparison of two taxa. The curves represent Loess-fitted regression curves.

A relaxation of constraint relative to null expectations is observed at the base of Synapsida, followed by their strengthening within individual lineages: caseids, eupelycosaurs, and sphenacodontians (within Eupelycosauria) (Fig. 3a). Constraints strengthen at the base of Reptilia but become relaxed around the earliest divergences of Diapsida. Subsequent strengthening of constraints is observed within the mycterosaurine varanopids and parareptiles. The overall pattern of relaxed constraints and elevated rates during early amniote evolution, followed by slowdowns and increased constraints, is consistent with models proposed by Simpson (1953) for phenotypic evolution during adaptive radiations: rapid evolution between peaks in the adaptive landscape and the lineages diverge into different regions of ecospace, followed by subsequent reductions in rate as niches are filled and constraint increases within the adaptive optima.

Constraints played a more important role during early reptile evolution than in early synapsids. Although both groups experienced initially relaxed constraints early in their evolution, reptiles reached character state saturation before the end of the Cisuralian; evidenced by asymptoting of the relationship between patristic distance and morphological dissimilarity (Fig 3b and Table 1). Synapsids, on the other hand, did not reach character state saturation and were, therefore, continuing to explore new areas of morphospace (Fig. 3b). Although the interquartile range and media of the morphological dissimilarities between taxa are similar for reptiles and synapsids, the maximum dissimilarities observed are considerably higher in synapsids, indicating their access to a larger area of morphospace (Fig. 3c).

**Table 1.** Estimates of morphological distance at the point of character state saturation (observed $V_{\max}$ parameter), compared with values expected from null simulations

Taxon subset	Observed *V*$_{\max}$	Null *V*$_{\max}$ values obtained from simulation, median (range)
Reptiles	0.60	0.81 (0.77–0.87)
Synapsids	1.12	0.81 (0.76–0.87)
Small amniotes	0.42	0.80 (0.75–0.87)
Medium amniotes	0.56	0.81 (0.76–0.88)
Large amniotes	0.61	0.83 (0.79–0.87)
Very large amniotes	0.63	0.81 (0.77–0.84)
Large and very large reptiles	1.00	0.80 (0.75–0.88)
Large and very large synapsids	1.03	0.81 (0.77–0.86)

### Variation between Anatomical Partitions

**Figure 4.**
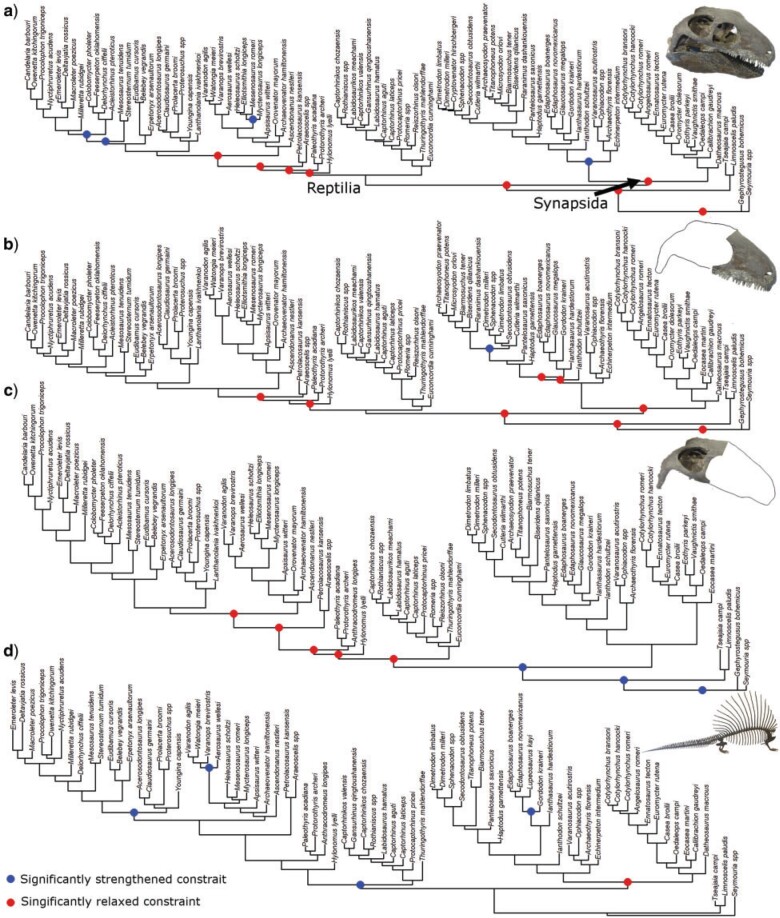
Patterns of constraint in amniotes within different anatomical partitions ploted over phlogenies where branch lengths represent the amount of character change within the character partition along that branch. Coloured nodes indicate significant strengthening or relaxation of constraints within clade. (a) Skull; (b) snout; (c) temporal region; and (d) postcranium.

**Figure 5.**
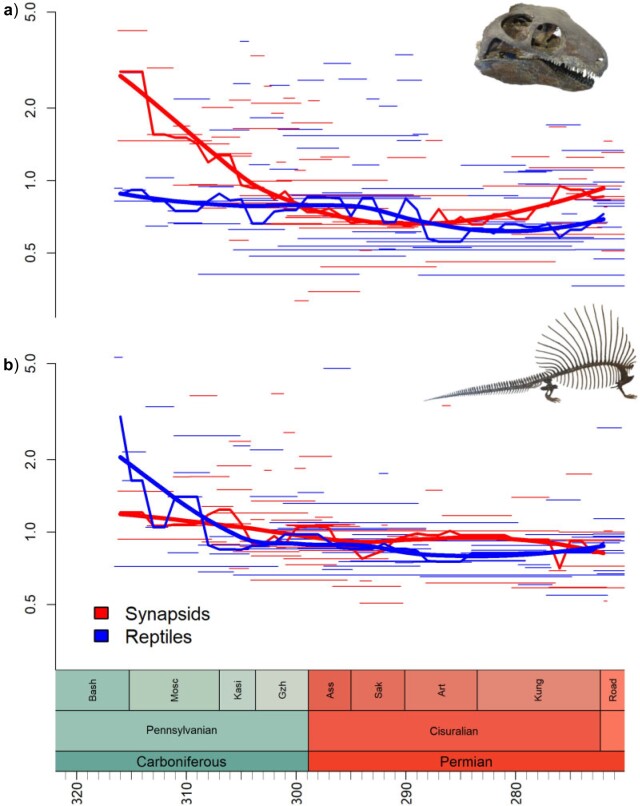
Rates of evolution in reptiles and synapsids within anatomical partitions through time. Narrow lines represent the rates of individual branches. The mid-weight line represents the median rate of each 1 million year time slice. The thick line represents Loess-fitted regression between median rate and time. (a) Skull and (b) postcranium.

Reptiles and synapsids differ in how patterns of evolution are expressed among different anatomical partitions. Both lineages exhibit relaxed constraints on skull evolution, in particular for the snout (Fig. 4a and b). In contrast, constraints in the temporal region of the skull, which houses the jaw-closing muscles and signifies important functional variation, are significantly relaxed in reptiles but not synapsids (Fig. 4c). This gave rise to the higher median and maximum values of morphological dissimilarity in this region among reptiles (Fig. S3c of the Supplementary material available on Dryad). Synapsids experienced elevated rates of skull evolution during their earliest evolution, which declined through the late Carboniferous before recovering slightly during the late Cisuralian (Fig. 5a). In contrast, rates of skull evolution in reptiles remained low throughout the study interval.

Reptiles show high early rates of postcranial evolution compared with those of synapsids (Fig. 5b). Nevertheless, postcranial rates declined through time and exhibit significantly high constraints in reptiles compared with synapsids, suggesting that high early rates did not result in the proliferation of a wide range of postcranial morphologies in reptiles (Fig. 4d). Synapsids experienced a significant relaxation of constraints on postcranial evolution (Fig. 4d), suggesting that although they evolved more slowly, they potentially acquired a wider disparity of postcranial morphologies (Fig. S3d of the Supplementary material available on Dryad).

Dental traits show an early release of constraints in both reptiles and synapsids. However, whereas reptiles retained these relaxed constraints within Diapsida and Neoreptilia, synapsids experienced a strengthening of constraints within eupelycosaurs (Fig. S3a of the Supplementary material available on Dryad). This was confirmed in our analyses of the larger dental dataset of Brocklehurst and Benson (2021), which could be analyzed over a longer study interval and provides evidence for a subsequent relaxation of constraints on dental evolution in the synapsid subgroup Therapsida (Fig. S3b of the Supplementary material available on Dryad).

### Constraint at Different Body Sizes

**Figure 6.**
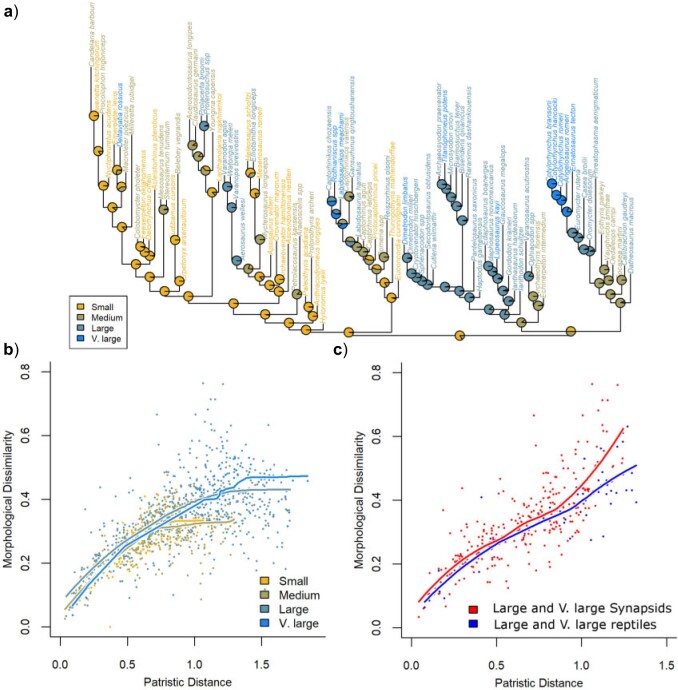
(a) Body size category assigned to each taxon, and likelihood ancestral state reconstruction of body size categories over the time-calibrated tree. The color of the tip represents the size assigned to that tip. Pie charts at each node represent the relative probability of each size category being the ancestral state of that node. (b) Patterns of constraint within each size category. Each point represents a pairwise comparison of two taxa within a size category. The curves represent Loess-fitted regression curve. (c) Patterns of constraint within reptiles and synapsids are assigned to the large or very large categories.

Small- and medium-sized amniotes were found to evolve under greater constraint than large and very large taxa, reaching character state saturation at a lower morphological dissimilarity (Fig. 6b). Interestingly, taxon pairs within all three size classes are found to evolve under greater constraints than expected from null simulations (Table 1). This suggests that morphological innovation among early amniotes was predominantly associated with evolutionary changes between size categories. Overall, therefore, taxa within different size classes occupy different regions of morphospace, with larger taxa occupying larger regions of morphospace (Fig. S6 of the Supplementary material available on Dryad).

**Figure 7.**
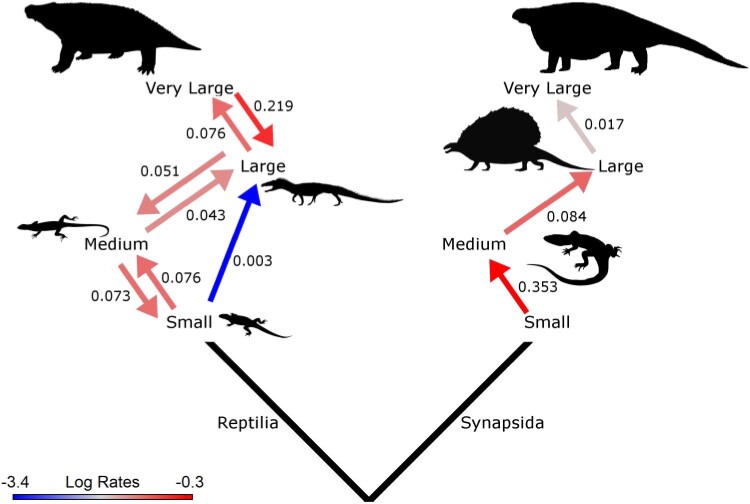
Rates of body size evolution in synapsids and reptiles. Numbers alongside arrows represent the rate of transition between the size categories (the instantaneous probability of transition) in the direction indicated by the arrow. Transitions with a rate of 0 are not shown. The color of the arrow represents the log-transformed rates. Silhouettes open source from phylopic.org (not to scale)

Although reptiles and synapsids show different patterns of body size evolution (see below), they nevertheless show similar patterns of overall morphological constraint within the size categories. Large and very large taxa exhibit relaxed constraints in both synapsids and reptiles, although early synapsids include more taxa of these sizes than do reptiles (Fig. 7c and Table 1).

We also find evidence for different patterns of body size evolution among synapsids compared with those in reptiles (see also Modesto et al. 2015; Brocklehurst 2021). The evolution of the discrete character representing body size best fits a model where the two lineages are subject to different evolutionary regimes (Table 2). Within synapsids, only transitions from smaller size categories to larger have positive rates; rates of transition from larger size categories to smaller were all zero (Fig. 7). This indicates an evolutionary trend toward larger body sizes in synapsids. Reptiles, on the other hand, have positive rate values for transitions in both directions, and in some cases, the rate of transition from larger to smaller size categories is higher than from smaller to larger.

**Table 2.** Results of likelihood fitting of models of discrete character evolution to the body size data

Model	Log likelihood	Akaike information criterion	Akaike weights
Single regime, equal rates	–109.52	221.04	$3.64 \times 10^{-19}$
Single regime, all rates are different	–91.31	214.73	0.017
Multi regime, equal rates	–107.74	219.50	$7.26 \times 10^{-4}$
Multi regime, all rates are different	–83.36	206.62	0.98

## Discussion

The evolution of full terrestriality, at the origin of amniotes, was a major event in vertebrate evolution, providing insights into macroevolutionary patterns during ecospace invasion. Moreover, the evolutionary divergences among early amniotes gave rise to the mammal-line (Synapsida) and reptile-line (Reptilia), which persist to the present day and show stark differences in their morphology, ecology, and biology. Previous studies indicated variation in rates and constraints coinciding with either the origin of amniotes or their subsequent diversification into different areas of terrestrial ecospace. This has been shown, for example, during body size evolution (Laurin 2004; Reisz and Fröbisch 2014; Brocklehurst 2016; Brocklehurst and Brink 2017; Brocklehurst and Fröbisch 2018; Brocklehurst et al. 2020), and the evolution of jaw (Anderson et al. 2013), tooth (Brocklehurst and Benson 2021), and limb morphology (Ruta et al. 2018). However, macroevolutionary patterns during this transition have been unclear due to the variation in taxonomic scope of these studies and regions of anatomy analyzed. Our study represents the first analysis that samples broadly across early members of the amniote crown-group and includes morphological variation from across the whole skeleton.

We find evidence for an early episode of high evolutionary rates across the skeleton, coupled with relaxed constraints on cranial (especially snout and dental) evolution. This is consistent with the early origins of a wide set of morphologically and ecologically distinctive amniote subclades by the late Carboniferous, including herbivorous edaphosaurids (Sues and Reisz 1998), macropredatory sphenacodontians (Fröbisch et al. 2011; Brocklehurst and Brink 2017), arboreal protorothyridids (Mann et al. 2021), and many other groups. These findings contradict those of Ruta et al. (2006), who suggested that amniote origins did not give rise to an increase in the rate of character state evolution. This may be an artifact resulting from under-sampling of amniote taxa and characters in the dataset of Ruta et al. (2006), who included only a very small sample of crown amniotes, from the reptile line only. Nevertheless, future studies should examine whether the elevated rates and subsequent decline observed here in early amniotes merely represent a subset of a longer-term decline in rates across tetrapods.

Our findings of relaxed constraints on cranial, snout, and dental evolution in the earliest amniotes are consistent with the hypothesis that diet-related cranial variation was an important axis of phenotypic diversification during their initial radiation (e.g., Janis and Keller 2001; Anderson et al. 2013; Brocklehurst and Benson 2021). We also show that rates of evolution were also elevated for other anatomical regions, not strictly limited to dietary or craniodental diversification. Nevertheless, those regions (postcrania, and the temporal region of the skull) exhibit different patterns of variation in constraint between reptiles and synapsids (Fig. 4), highlighting the divergent paths to morphological diversification that were taken by these groups.

The initial diversification of reptiles appears to have been focused on the temporal region. This is consistent with the qualitative observation that early reptiles exhibit considerable evolutionary versatility of temporal fenestration, contrasting with more conserved temporal anatomy in synapsids (Piñeiro et al. 2012; MacDougall and Reisz 2014; Haridy et al. 2016; Ford and Benson 2020). Diversification of temporal fenestration among early reptile groups likely corresponds to variation in muscle attachment and jaw function (Frazzetta 1968; Werneburg 2019; Abel and Werneburg 2021) and may therefore reflect an early diversification of cranial function. Reptiles also maintain relaxed constraints on their dentition, whereas eupelycosaurian synapsids experience a strengthening of these constraints. This may result from differences in the evolvability of palatal dentition between synapsids and early reptiles. Reptiles exhibit great diversity in the arrangement, size, density, and patterns of loss of the palatal dentition, both today and in the past (Matsumoto and Evans 2017). Such variation is also present, to some extent, in caseasaurian synapsids (Brocklehurst et al. 2016). However, eupelycosaurian synapsids show much less variability, with a trend to simplification and ultimately loss of palatal teeth long before the origin of mammals (Matsumoto and Evans 2017). This may have been compensated by a relaxation of constraints on the marginal dentition along the line leading to mammals, which is evident among the middle Permian divergences of therapsids (Fig. S3b of the Supplementary material available on Dryad). Increases in the evolutionary versatility of marginal dentition among synapsids culminated in the development of strongly heterodont and functionally differentiated marginal dentitions as a central innovation of later cynodonts, including mammals (Compton and Jenkins 1968; Luo et al. 2015).

Postcranial data indicate a decoupling of rates and constraints. Reptiles show high early rates coupled with significantly increased constraint suggesting that they rapidly explored a relatively small postcranial character state space. In contrast, early synapsid postcrania evolved at lower rates for much of the Carboniferous and early Permian, but under significantly relaxed constraints that allowed them to gradually explore a much larger character state space. Our findings are therefore potentially consistent with the limited previous studies of synapsid postcranial variation, which found evidence for the high disparity of humerus shape and heterogeneity between vertebral regions in therapsids (Jones et al. 2018; Lungmus and Angielczyk 2021).

Synapsid evolution is further distinct from that of reptiles in that synapsids rapidly attained large body sizes during their early history, whereas reptiles did not. Ancestral character state mapping suggests a small-bodied ancestor of the amniote crown-group ($<$1 kg) (Fig. 7a), from which multiple synapsid lineages independently evolved large body sizes exceeding 40 kg before the end of the Carboniferous (Reisz and Fröbisch 2014; Brocklehurst and Brink 2017; Brocklehurst and Fröbisch 2018). In contrast, reptiles did not reach such sizes until the latest Cisuralian origin of moradisaurine captorhinids (Brocklehurst 2016). The analysis of body size evolution presented here should be treated with caution due to the low resolution of the size data, but it does demonstrate that synapsid body size was evolving under a distinct regime to that of reptiles and potentially exhibited a trend of increasing body size that was absent in reptiles.

It has been suggested that the apparent increase in body size may have driven the apparent greater diversity of synapsids during the Paleozoic, due to biases in either preservation or collection: larger synapsids may be easier to find or have greater fossilization potential than smaller, more fragmentary reptiles (Modesto et al. 2015; Brocklehurst 2021). The fossil reptiles named from the Carboniferous are smaller but more complete than the synapsids (Modesto et al. 2015). This potentially indicates that only the most complete reptiles are collected or described, whereas synapsid material may be considered informative even when it is more fragmentary, due to the larger body size of synapsids (Modesto et al. 2015). However, it is also possible that this signal is genuine and reflects the earlier adoption of herbivory and carnivory in synapsids (Modesto et al. 2015), reflecting a wider pattern of divergence along ecological lines in early amniotes.

Irrespective of patterns of species diversification, larger body size appears to be related to reduced constraint in morphological evolution, both in reptiles and synapsids (Fig. 6b and c). Therefore, the fact that synapsids show an early trend toward larger body size, reaching larger sizes earlier and more frequently than the reptiles, may have permitted the greater relaxation of constraints observed in early synapsids. A greater range of body sizes, as observed in synapsids, has been linked to greater functional diversity (Woodward et al. 2005; Rooney and McCann 2012). Larger body sizes allow access to a different range of ecotypes, including macro-predation and high fiber herbivory (Clauss and Hummel 2005; Müller et al. 2013; Brocklehurst and Brink 2017), permitting further diversification within these distinct regions of ecomorphospace. Moreover, the fact that synapsids rarely underwent reversals to small body sizes could result in fewer homoplasies among size-dependent characters and, therefore, weaker apparent constraints on morphological evolution.

Early attainment of large body size is particularly relevant to the release in constraints on postcranial evolution observed in synapsids, but not in reptiles. Scaling relationships imply that the stresses experienced by the skeleton are relatively higher in larger-bodied species (Stanley 1973; Biewener 1982); thereby, ecological specialization may require more substantial postcranial specialization among large-bodied species to exploit new niches. Studies in mammals support these observations, demonstrating greater diversity within different locomotor modes at larger sizes (Weaver and Grossnickle 2020) and greater distinction between locomotor types in larger taxa (Jenkins 1974; Jenkins and Parrington 1976; Runestad and Ruff 1995). The relaxed evolutionary constraints in the synapsid postcranium may provide an explanation of why large body size evolved multiple times independently among early synapsids, but not among early reptiles; synapsids’ access to a wider region of postcranial morphospace allowed the greater postcranial specialization necessary for ecological specialisation at large sizes. Alternatively, large body size may have evolved in synapsids for other reasons (e.g., ecological), and necessitated postcranial specializations that are detected here as a release in constraint on postcranial evolution.

Early events in amniote evolution, documented here, set the stage for the origins of major groups that comprise most of the extant diversity of land vertebrates. In particular, Neodiapsida (including the reptile crown-group; “Neo” in Fig. 1) and Therapsida (including the mammalian crown-group; “The” in Fig. 1) comprise the bulk of amniote diversity after the early Permian and have highly distinct anatomy compared with their predecessors (Rubidge and Sidor 2001). These groups evolved substantial morphological disparity (Ruta et al. 2013a, 2013b; Ezcurra and Butler 2018; Grunert et al. 2019; Lungmus and Angielczyk 2021). However, the branches leading to them, which span most of our early Permian study interval, show no evidence of high rates of evolution. In fact, both lineages represent evolutionary slowdowns relative to the “backbones” of the reptile and synapsid phylogenies, demonstrating that high evolutionary rates are not required to explain the origins of these groups. This raises the possibility that distinctive traits of both neodiapsids and therapsids assembled gradually throughout the early Permian (Cisuralian) during a cryptic and poorly sampled interval of evolutionary history, before their rise to high abundances during latter intervals of Permian and the Triassic. However, both groups exhibit a long interval in which direct evidence for rates of accumulation of their derived characters is entirely missing the fossil record (late Pennsylvanian–latest Cisuralian). Therefore, future fossil discoveries are required to test hypotheses of their evolutionary patterns and could demonstrate, in reality, their traits appeared abruptly, either late or early in this unsampled time window. This is particularly relevant to discussions on the origin of therapsids, where it has been suggested that the interval known as Olson’s Gap (late Kungurian-Roadian, latest Cisuralian-earliest Guadalupian) (Lucas and Heckert 2001; Liu et al. 2009; Brocklehurst 2020) was an important evolutionary interval and that better sampling at this time would shed light on the origins of therapsid anatomy (Abdala et al. 2008; Liu et al. 2009). Our results show that current data are also consistent with a protracted origin and that a great expansion of fossil evidence throughout the early Permian could be required to fully test this.

Variation between macroevolutionary patterns among different anatomical partitions for early synapsids and early reptiles may have underpinned the different evolutionary trajectories of reptile- and mammal-line amniotes and may ultimately have resulted in the clear disparities between mammals and reptiles today. Our results potentially imply that a deep divergence in patterns of evolutionary modularity (Vermeij 1973; Wagner and Altenberg 1996), due to either developmental or ecofunctional drivers might potentially explain the different paths that reptiles and synapsids have taken during their ecological diversification. For example, we provide evidence for the relaxation of constraints on temporal evolution among early reptiles (Fig. 4). This is congruent with variation seen among extant reptiles, which exhibit substantial disparity of temporal morphology and of regions associated with jaws muscular and articulation (Watanabe et al. 2019; Rhoda et al. 2021), as well as variation in the degree of cranial kinesis and the location of articulation points. This ranges from relatively akinetic skulls in crocodiles, turtles, and rhynchocephalians (Preuschoft and Witzel 2002; Ferreira et al. 2020) to mobility of the frontoparietal suture, quadrate, and palate in many squamates, extreme forms of kinesis in snakes (Metzger 2002; Rhoda et al. 2021), and kinesis of the beak relative to the braincase in birds (Bout and Zweers 2001). Variability in kinesis and articulation is noticeably more limited, or absent, in mammals.

Relaxation of postcranial evolution is shown here among early synapsids (Fig. 4). This is congruent with considerable variation in the postcranial morphology of modern mammals and their therapsid ancestors, including greater variability and distinction between modules in the vertebral column in mammals compared with reptiles (Arnold et al. 2017; Jones et al. 2018; Arnold 2021) and considerable increases in synapsid humeral disparity from the middle Permian onwards (Lungmus and Angielczyk 2021). Our findings, therefore, suggest that an initial release in postcranial evolution occurred at the origin of synapsids, with subsequent increases occurring in therapsids and among mammals (Jones et al. 2018; Lungmus and Angielczyk 2021).

## Conclusion

Synapsids and reptiles in the present day represent two highly divergent lineages, with substantial differences in their morphology and physiology. During their earliest divergences in the Carboniferous, however, they were morphologically and ecologically very similar, both represented by small insectivores with a superficially “lizard-like” body form. These superficial similarities mask a deep evolutionary divergence in rates and modes between synapsids and reptiles, documented by our analyses. Analysis of constraint without *a priori* assumptions on where regime shifts occur identifies fundamental differences in patterns of evolution between the two lineages: reduced constraint in the temporal region of the skull on the reptile line and reduced constraints in postcranial evolution on the synapsid line, which may potentially be linked to a trend towards increasing body size. This separation of evolutionary patterns during the earliest divergence between synapsids and reptiles may be fundamental to understanding the differences between these groups throughout their history, including stark differences between members of these groups that are present today. Our observations of a deep divergence of macroevolutionary modalities raises the possibility of a deep divergence of either developmental processes or ecological factors very early on the mammal and reptile lines. Further studies of morphological evolution spanning the subsequent intervals of amniote evolution are required to confirm this possibility. Moreover, developmental and ecological or functional studies are also required to test the mechanisms that may have given rise to this macroevolutionary divergence. Amniote origins and the long-term differentiation of mammal and reptile phenotypes, therefore, provide a promising avenue for cross-disciplinary investigation in evolutionary research.

## Supplementary Material

Data available from the Dryad Digital Repository: http://dx.doi.org/10.5061/dryad.9ghx3ffj5.
